# Supplementary figures and images for: PyClone-VI: scalable inference of clonal population structures using whole genome data
Source: BMC Bioinformatics. 2020 Dec 10;21:571. doi: 10.1186/s12859-020-03919-2 (PMC7730797; doi:10.1186/s12859-020-03919-2)

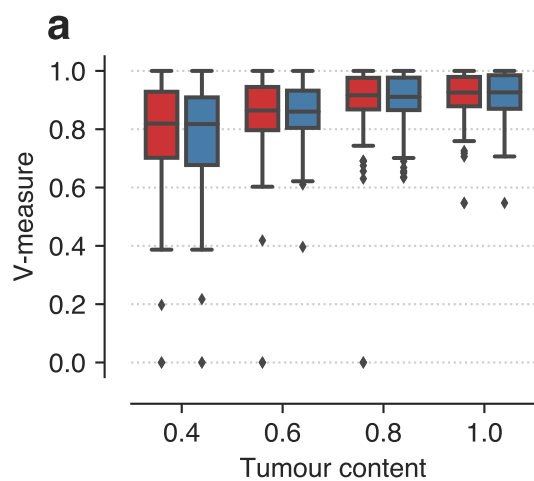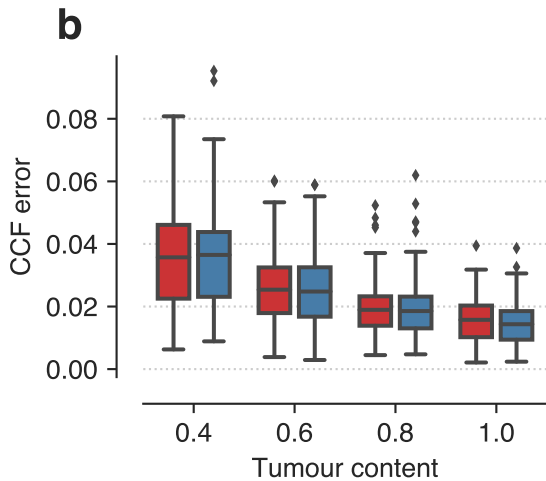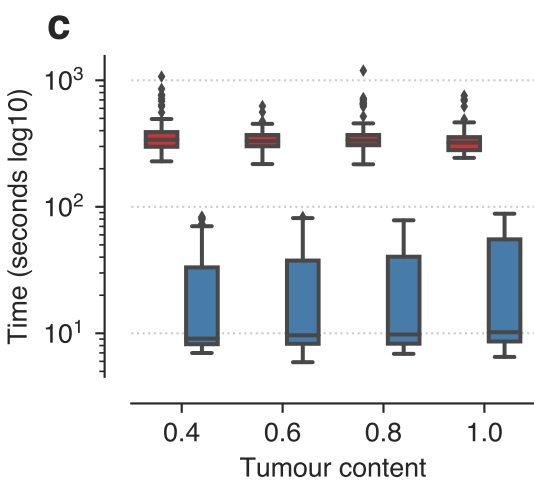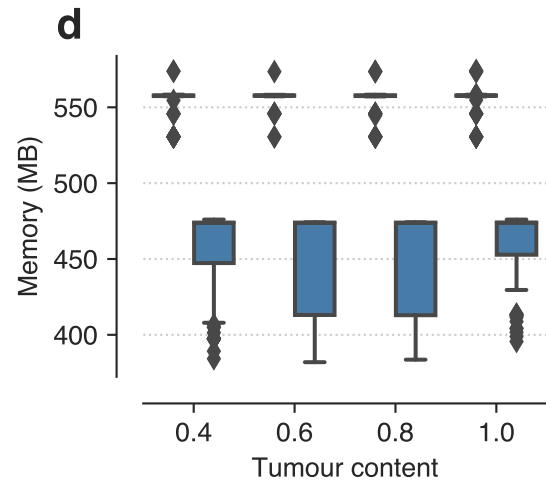

PyClone PyClone-VI

Supplement: Supplementary file 2 — Additional file 2. Fig. S1: Comparison of PyClone and PyClone-VI with varying tumour content PDF file with figures showing the results of running PyClone and PyClone-VI with varying tumour content values. Data was simulated from the PyClone model with 4 samples, 100 mutations, a mean depth of 100 reads and copy number ranging from 1–4 copies. The same tumour content values were used for all 4 samples for each dataset. a V-measure as a function of the tumour content of the samples. b Mean absolute deviation of inferred CCF from truth as a function of the tumour content. c Runtime of the methods. d Memory usage. [file 12859_2020_3919_MOESM2_ESM.pdf]

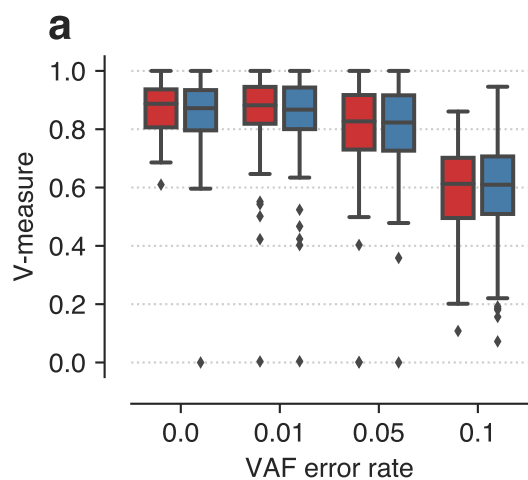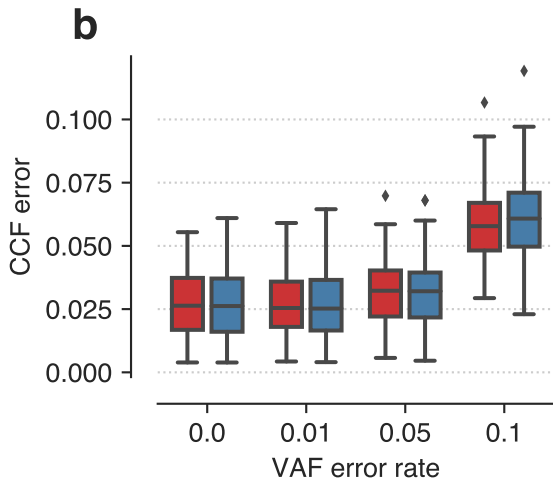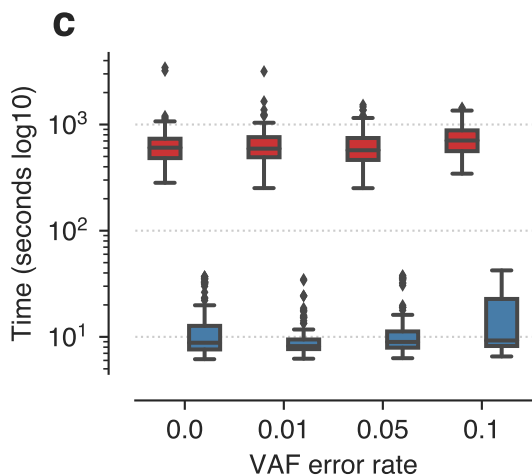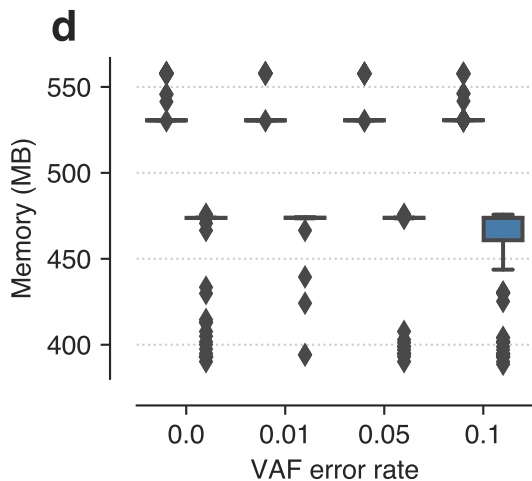

PyClone PyClone-VI

Supplement: Supplementary file 3 — Additional file 3. Fig. S2: Comparison of PyClone and PyClone-VI with varying error rates PDF file with figures showing the results of running PyClone and PyClone-VI with varying the error rates of the expected variant allele frequency. Data was simulated from the PyClone model with 4 samples, 100 mutations, a mean depth of 100 reads, copy number ranging from 1–4 copies and tumour content for each sample randomly selected from [0.4, 0.8]. To simulate error, the true expected VAF f was computed for each mutation and then a perturbed expected VAF $\tilde{f}$f~ was simulated uniformly from $[max\{0, f - \epsilon \}, min\{1, f + \epsilon \}]$[max{0,f-ϵ},min{1,f+ϵ}] where $\epsilon$ϵ is the error rate. The perturbed expected VAF $\tilde{f}$f~ was then used to simulate read count data. a V-measure as a function of the error rate. b Mean absolute deviation of inferred CCF from truth as a function of the error rate. c Runtime of the methods. d Memory usage. [file 12859_2020_3919_MOESM3_ESM.pdf]

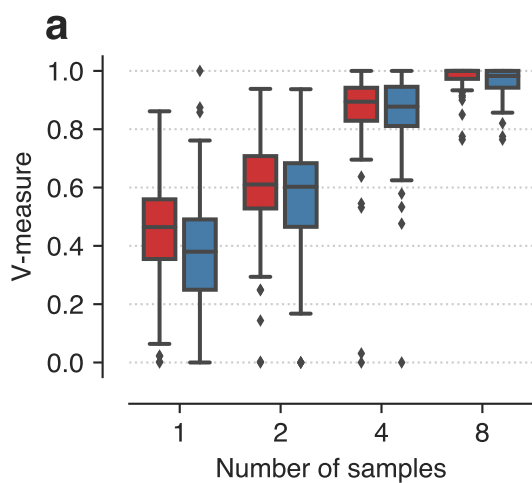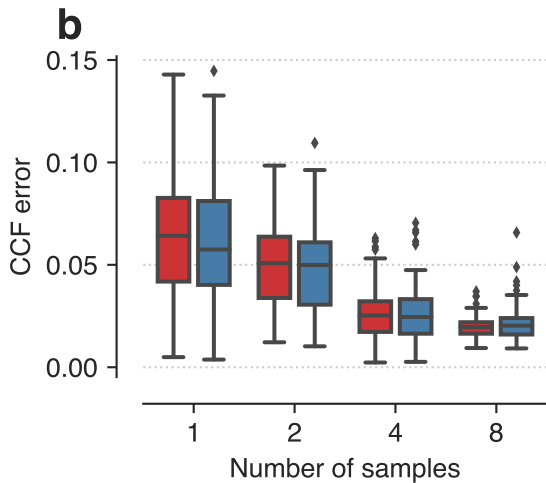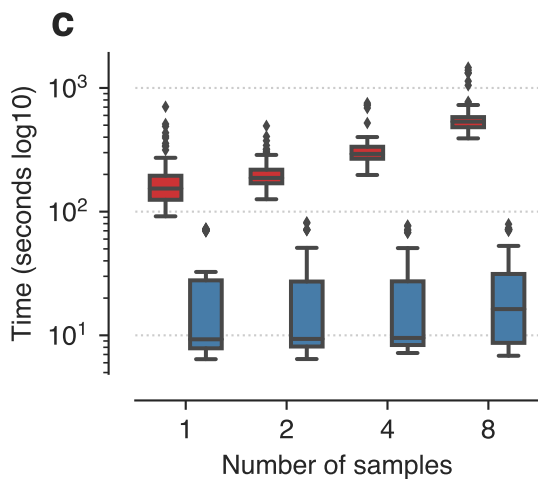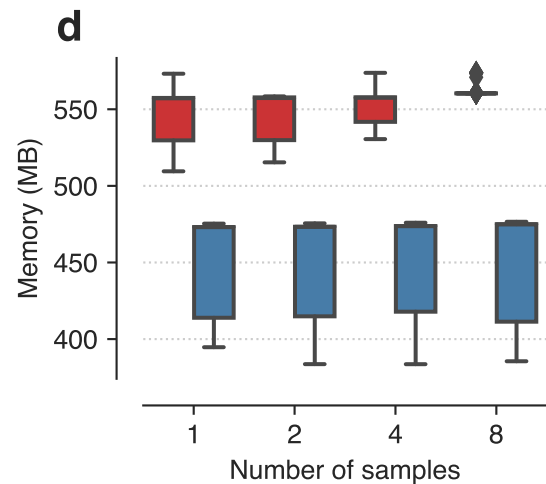

PyClone PyClone-VI

Supplement: Supplementary file 4 — Additional file 4. Fig. S3: Comparison of PyClone and PyClone-VI with varying number of samples PDF file with figures showing the results of running PyClone and PyClone-VI with varying number of samples. Data was simulated from the PyClone model with 1–8 samples, 100 mutations, a mean depth of 100 reads, copy number ranging from 1–4 copies and tumour content for each sample randomly selected from [0.4, 0.8]. a V-measure as a function of the number of samples. b Mean absolute deviation of inferred CCF from truth as a function of the number of samples. c Runtime of the methods. d Memory usage. [file 12859_2020_3919_MOESM4_ESM.pdf]
